# Combatting intergenerational effects of psychotrauma with multifamily therapy

**DOI:** 10.3389/fpsyt.2022.867305

**Published:** 2023-02-01

**Authors:** Trudy Mooren, Elisa van Ee, Irma Hein, Julia Bala

**Affiliations:** ^1^ARQ National Psychotrauma Centre, Diemen, Netherlands; ^2^Department of Clinical Psychology, Faculty of Social Sciences, Utrecht University, Utrecht, Netherlands; ^3^Psychotraumacentrum Zuid-Nederland, Reinier van Arkel, ‘s-Hertogenbosch, Netherlands; ^4^Behavioural Science Institute, Radboud University, Nijmegen, Netherlands; ^5^Department of Child and Youth Psychiatry, Amsterdam Academic Medical Center, Amsterdam, Netherlands; ^6^Consultant, Diemen, Netherlands

**Keywords:** intergenerational, psychotrauma, refugees, veterans, multi-family treatment

## Abstract

There is growing evidence that parental trauma is associated with psychosocial disorders, externalizing and internalizing problems, and higher sensitivity to posttraumatic stress disorder (PTSD) in children. Recent research findings suggest multidimensional relational, psychological, and neurobiological interrelated pathways of intergenerational influence. Moreover, the intergenerational effects of parental trauma need to be understood within a broader systemic context, as a part of family adaptation. This article explores research findings and clinical practice to enhance our understanding of intergenerational processes and presents directions for therapeutic interventions. A trauma-focused multi-family therapy, aiming to restrict the relational consequences of parental trauma and strengthen family resilience, is described. The proposition is that to facilitate and improve the quality of parent–child interaction in response to psychotrauma, fostering emotion regulation capacities and mentalization is crucial. These efforts offered through family group interventions may benefit various families coping with adversity in culturally diverse societies.

## Introduction

The search for mechanisms of intergenerational consequences of psychotrauma that started with clinical observations of children of survivors of the Holocaust (1, 2) has a long history characterized by many controversies. Despite decades of research the mechanisms of intergenerational consequences of trauma remained insufficiently explained and lacked a strong empirical basis (3–5). The underlying premise, based on a linear cause-effect model, is that parental trauma is directly or indirectly passed down to the offspring. What is transmitted, through which processes, leading to which consequences has been a subject of long and at times heated debates. One of the first attempts to clarify controversies about the transmission of trauma emerged from a meta-analytical study of secondary traumatization of the children of survivors of the Holocaust (6). In a set of controlled non-clinical studies, no evidence for the influence of the parents’ traumatic Holocaust experiences on the wellbeing of their children was found in the general population. Therefore, the conclusion was that secondary traumatization only emerged in studies with clinical participants.

In the past decades, research expanded, and studies included clinical populations, offspring of the survivors of the Holocaust, combat veterans, traumatized refugees, victims of community violence, and interpersonal trauma. These enhanced our understanding of the impact of parental PTSD on children, parent–child relations, and parenting. In Table 1, factors explaining the impact of parental PTSD on their offspring are summarized. These factors are based on recently published reviews and meta-analyses on various traumatized populations.

**TABLE 1:** Mechanisms of parental PTSD on offspring mental health (reviews).

References	Study population	Country	N included studies	Period	N participants	Outcomes	Theory	Analyses	Mechanism	Directions for research
Creech and Misca 7	Military and veterans	United States	20	2001–2016	4727 parent-child dyads, plus 272 individual children	Parenting behavior, children’s outcomes	Cognitive behavioral interpersonal	Narrative	Higher experiential avoidance was associated with less positive engagement with children; Perceived parental difficulties; Increased parenting stress	Research in non- US population Standardized measures of child outcomes Representation of women in samples; Longitudinal design
Dashorst et al. 12	Holocaust offspring	Various, US, Europe, and Israel	23	2001–2015	2981	Mental health outcomes in offspring	Intergenerational traumatization; attachment; Diathesis-stress model; Biological, and epi-genetical changes	Narrative	Parental mental health problems, in particular PTSD is related to PTSD and depressive symptoms in offspring; Having two survivor parents; Parental gender (mothers more associated with “transmission”). Additional stress and life events, psychophysiological processes, perceived parenting, and attachment: HSO families characterized by relatively many or intense conflicts within families and less cohesion; Changed cortisol levels, increased methylation in specific gene- segments.	Multiple factors, rather than one single factor determines mental health outcomes in offspring; Longitudinal design
Dekel and Goldblatt 3	Veterans	Various, mostly American/Vietnam	15	1998–2004	3188 Father-son relations	Parent-child relationship	Secondary traumatization; Psychodynamic process: projection and identification, difficulties containing emotions; Family dynamics, and including communication	Narrative	Father’s trauma and distress: the greater the father’s distress, as expressed in severe PTSD, and the more frequent the use of violence, the greater the extent of the children’s distress	Not only focus on fathers’ PTSD, but also co-morbid symptoms, e.g., addictive behavior, adopt a broad perspective, including factors that mitigate distress or transmission
Flanagan et al. 13	Refugees	Europe, USA	8	2006–2018	1684	Individual offspring	Intergenerational consequences of PTSD symptoms, attachment	Narrative	Disrupted attachment, maladaptive parenting style, diminished emotional availability, disrupted family functioning, accumulaton of family stressors, dysfunctional communication styles, and severity of parental symptomatology	Prospective, longitudinal design, using culturally appropriate measures.
Hope et al. 8	Various, military, interpersonal violence, birth experiences, and unspecified	Various	27	2004–2017	9117 parents, parent-child dyads	Parenting	Consequences of PTSD symptoms on parenting, in terms of relationship quality (with child), parenting style, satisfaction, and parenting stress	Narrative	Parental PTSD associated with elevated levels of parenting stress, detrimental effects to parenting satisfaction, the parent-child relationship and endorsement of negative parenting practices (less sensitive, more controlling, aggressive parenting, and inconsistent).	Substantial sample sizes Inclusion of fathers. Representation of types of trauma in light of generalizability.
Kritikos et al. 14	Military or veteran	Various, e.g., Balkan, Vietnam, and Lebanon	28	1993–2014	3684 parents, or offspring	Parenting problems, family adjustment and offspring problems	System theory, social interaction theory	Meta-analysis	Small to moderate effect sizes found for relationships between parental PTSD/S and family difficulties, parenting problems and poor family functioning. Design of study, informant of child outcomes, and type of child outcomes moderated the relationship between PTSD/PTSS and family difficulties.	Evaluate moderators that explain heterogeneity in offspring response to parental PTSD/PTSS. Longitudinal design. Include multi-informant measures to compare effects by reporter.
Van Ee et al. 10	Various, child abuse, childbirth, combat, interpersonal violence, mass violence, natural disaster, prematurity, and stillbirth	Various	72	1992–2014	14109 parents or offspring	Parent-child relationship, parenting, and impact on the child	Relational and transactional perspective; attachment and mentalization, and the cycle of abuse	Narrative	Parents with PTSD are emotionally less available and perceive their children more negatively than parents without symptoms of PTSD; Children of parents with PTSD are at a younger age more easily deregulated or distressed and at an older age facing more difficulties in their psychosocial development than children of parents without PTSD.	Include a framework of resilience. Sound measurements of PTSD, uniformity in used concepts and standardized observational measurements, and longitudinal studies are needed.

Shown in alphabetical order, references (7, 8, 12, 3, 13, 14, and 10).

Different reviews on the impact of parental PTSD on parenting suggest that PTSD affects parenting, parent–child relationships, and child outcomes (7–10). A meta-analytic study confirmed the association of parental trauma with children’s distress and behavioral problems (11). Furthermore, several relational patterns and various pathways of the impact of parental trauma on offspring were identified. Alongside the growing evidence of relational pathways through which parental trauma affects children, more insight into neurobiological mechanisms underlying these pathways has emerged from research on brain functioning, neuroendocrinology, and (epi)genetics (12).

The intergenerational effects of PTSD and comorbid psychopathology are not limited to the offspring. A systemic review of literature (15) revealed that not only does PTSD affect the relationships of those with the disorder, but PTSD itself is also affected by those relationships as well. There is thus a reciprocal, bi-directional association between PTSD and close relationships. The impact of parental trauma on children can be seen as a part of a dynamic systemic process of family adaptation. The trauma of one family member influences the whole family (16, 17). Intrusive experiences are followed by complex posttraumatic family adaptation and reorganization with functional or dysfunctional outcomes (18), depending on individual and family developmental phases and socio-political and cultural contexts. The positive outcome of successful family adaptation has received little attention for a long time, but now there is a growing tendency to focus on resilience and positive aspects of intergenerational dimensions of parental trauma (19, 20).

In this article, we will draw a conceptual framework, based on theoretical and clinical perspectives derived from the psychotrauma domain and the attachment field. Beyond the discourse on stabilization versus exposure which has dominated the trauma field for a while, we consider emotion regulation a transdiagnostic process, crucial in the aftermath of experiencing traumatic events. Insights from the literature on trauma exposure and on intimate interrelationships serve to better understand the risk and protective processes at various system levels in the aftermath of violence. Thereby enhancing possibilities for interventions that break the intergenerational transmission of trauma. We will shed light on the dynamics of emotion regulation and mentalizing as crucial elements of parent–child interaction and will describe how these integrated perspectives are used in well-studied interventions for alleviating consequences of psychotrauma in families confronted with intrusive adversity. As these processes may be different for age groups, we have chosen to focus specifically on families with children aged 0–5 years, where dependency is relatively large.

## Multiple pathways of intergenerational impact

**FIGURE 1:**
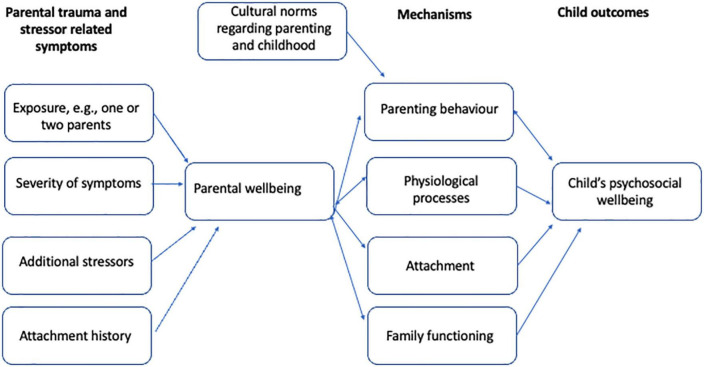
Mechanisms of intergenerational consequences of psychotrauma.

Recent reviews on the mechanisms delineating intergenerational traumatization in Holocaust stricken population, distinguished as plausible mechanisms: the severity of traumatization of parents, having one or both parents suffering from psychopathology, attachment and quality of parent–child relationship, and psychobiological alterations (12, 21). The burden of traumatic experiences, attachment and mentalization, and neurobiological and epi-genetical influences were also identified as key issues in reviews on relational patterns between traumatized parents with symptoms of PTSD and their non-exposed children (10, 13, 14). In the following, we will focus on attachment and related processes and biological and genetic influences as intergenerational mechanisms. A conceptual model has been depicted in Figure 1.

### Attachment, mentalization, and emotion regulation

Sensitivity and responsiveness of the parent are considered essential for a child’s development of a secure attachment. Mothers with more symptoms of PTSD, however, were observed to be less sensitive and responsive (22–25), more avoidant (26), more intrusive (27), and more overprotective (28). Parents with PTSD were also reported to be more hostile, rejective, or aggressive toward their children (29–31). Aggression and emotional withdrawal seem especially likely when the trauma of the parent is connected to the child, for instance, when the child has been born out of rape, eventually resulting in disorganized attachment styles (32). Besides abuse, disorganized attachment of the child could also be the result of neglect, failure to soothe and comfort the child, and thereby failure to regulate the child emotionally (33). Consequently, mothers and fathers who experienced trauma can have a less optimal attachment relationship with their children (34–36).

Mentalization is the capacity to perceive and understand mental states of the self and the other that help to explain and predict feelings, thoughts, and behavior (37). The capacity of the caregiver to mentalize, and then to respond to the child’s cues, is fundamentally supportive of the regulation of emotions and the development of mentalization competencies within the child (38). Maternal interpersonal violence-related PTSD and reflective functioning (as an operationalization of mentalization) were significantly associated with mothers’ mental representations of their young children (39). Mothers suffering from PTSD revealed the most difficulties with understanding the minds of their children. Indeed, a meta-analysis established a relation between PTSD symptoms and social-cognitive performance including mentalization (40).

### Biological and genetic influences

Twin studies have indicated that genetic factors influence exposure to potentially traumatic events and that those genetic influences explain a substantial proportion of vulnerability to PTSD (41). Research on possible candidate genes focuses on dopamine (DA) system genes and the serotonergic system (41). Neuroendocrine studies in offspring of parents with PTSD show that they have significantly lower plasma cortisol levels compared to offspring of survivors without PTSD (42). These neuroendocrine measures were negatively correlated with the severity of parental PTSD symptoms. Also, observations of infants born to mothers who were pregnant on 9/11 demonstrate that low cortisol in relation to parental PTSD was present early during development and may be influenced by *in utero* factors such as glucocorticoid programming (42). Since low cortisol levels are particularly associated with the presence of maternal PTSD, the findings suggest the involvement of epigenetic mechanisms (42). It is not clear whether epigenetic marks are equally stable across all genes and all gene regions since some epigenetic marks have been shown to persist across generations, while others have demonstrated a change in response to psychotherapeutic interventions (43).

## Resilient family adaptations

Members of a family respond to trauma and threat as a system in which both compensation and exacerbation of symptomatology are possible. Children in families of victims of torture, for example, can each adopt functional roles that differ in resiliency and vulnerability. Punamäki (44) describes: One sibling may be the “symptom carrier” who shows vulnerability and expresses pain, another is the “family psychologist” who takes care of, consoles, and encourages suffering members, and the third is the “sunshine” and savior child who makes others forget and compensates for the losses.

How a family confronts crises and challenges and reorganizes and reinvests in life will influence the adaptive abilities of all its members and their relationships (45). Resilience has become a dynamic, interactive, process-oriented concept (46–48). Individual resilience is best understood and fostered as a mutual interaction of the individual, family, sociocultural, and institutional influences (47, 49). Protective processes at the family level include family cohesion and flexibility, open, clear communication, rules and rituals, family meaning of adversity, problem-solving abilities and capacity to utilize external resources, and cultural traditions (45, 47, 50). Intergenerational transmission of resilience has received scarce attention within the literature but further delineates the complex processes between traumatized parents and their children.

Parenting cannot only buffer the effect of war and violence but also enhance the processes by which children become resilient despite the adverse context. In war, loving and non-punitive parenting was found to be associated with children’s high creativity and cognitive competence, which in turn could protect their mental health (51). Resilience in parents promotes resilience in children. Adult attachment representations may explain differences in post-trauma adjustment and parenting. A study of 53 asylum-seeking and refugee parents analyzed the relations between the number of traumatic experiences and symptoms of PTSD, parental sensitivity, and attachment representations (52). The results show that when parents are less able to rely on secure attachment representations, the number of reported traumatic experiences and symptoms of PTSD increase the risk of insensitive parenting. Because secure attachment representations serve to protect a child against the effect of parental trauma, these parents need to be supported in confirmation of secure models of the world and significant others, and the ability to offer a sensitive attachment relationship to the child.

### Communication as a core vehicle

Communication about the traumatic past may facilitate coming to terms with the past and improve attachment. Implicit communication, over-disclosure or silencing can interfere with functional posttraumatic family adaptation. Based on their review, Dalgaard and Montgomery (53) emphasized that the timing and strategy of disclosure by parents about the traumatic past matters most. The level of parental disclosure that promotes psychological adjustment in (refugee) children depends on whether the children themselves have been directly exposed to traumatic experiences, and whether the children are prepubescent or older (53). Moreover, the process of trauma disclosure is highly culturally embedded, and different favorable strategies may exist for different target groups.

## Pathways for intervention

**FIGURE 2:**
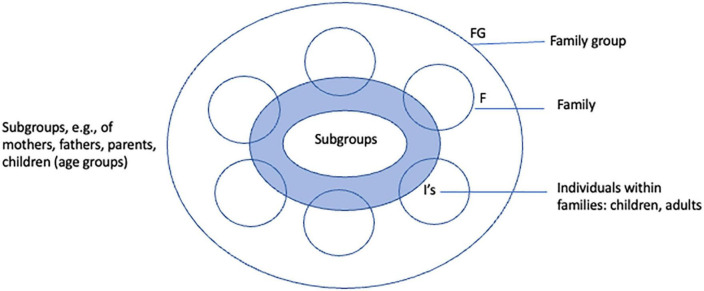
Multi-family framework.

Many of the traumatized parents present a complex array of difficulties and mental health problems. Therefore, a combination of techniques is often needed to alleviate the symptoms of stress and improve family interactions. Several treatment methods that focus on specific traumatized target groups are available. For instance, the program Families OverComing Under Stress (FOCUS) aims to support veteran families in the aftermath of deployment (54). Diamond and colleagues developed an attachment-based model for working with families with adolescents struggling with suicidality (55). Figley and Kiser offer a treatment program to traumatized families based on a family group format (56). Whereas differences among models exist, for instance, concerning specific target populations, communalities consist of a focus on setting and communicating goals, psycho-education, creating narratives (of what happened), and developing skills (emotion regulation, communication, and solving problems). Recently, upon drawing child trauma treatment models together, Kiser and colleagues emphasized that few studies explore the impact of parent/caregiver inclusion in child trauma treatment models. “There is a significant need for future studies on the impact and mechanisms of parent/caregiver trauma and the integration into child trauma treatment” [(57), p. 66]. As an example of a treatment approach including adults and children, we’ll continue to describe multifamily therapy (MFT). MFT programs for different groups of traumatized families (58, 59) such as refugees and veterans are multidimensional and focus on the interplay between internal (biological, psychological) and external (familial, cultural, social, political) influences that hinder or facilitate the development of children and families. MFT is an approach that encompasses a contextual setting enabling families to make changes through variations of flexible settings (family or parental group, parent–child dyads, subgroups of fathers, mothers, children, and individuals) (see Figure 2). Trauma-focused individual therapy for parents Eye Movement Desensitization Reprocessing (EMDR), Narrative Exposure Therapy (NET), Brief Eclectic Psychotherapy (BEPP), and preverbal EMDR for children can be integrated in the program simultaneously or successively. Systemic, mentalization-based, and behavioral interventions are planned on multiple system levels.

The treatment aims to reduce parental limitations evoked by PTSD, enhance sensitive parenting, and secure attachment. Mentalization, emotional regulation, and empowerment are used as the main pathways to reduce trauma-related intergenerational effects on parenting, parent–child relationships, and child development (60, 61).

### Mentalization: Enhancing reflexive parenting

Multifamily therapy for traumatized families relies on mentalization-based multi-family therapy, developed in Marlborough Family Service in London (62). It has been based on attachment theory (improving interpersonal relationship with a focus on mentalizing and systemic theories). Creating a context for exploring trauma-related influences that shape parental representations of a child, current attachment representations and parenting practices in interactions with cultural and familial influences, can be achieved in different ways. Parental group or subgroup discussions, mutual interviews or role-playing, modeling, and video feedback are used to enhance parents’ capacity for mentalizing. Reflection on thoughts and feelings during a parental withdrawal and its consequences for the child, for example, can take place in a moment of interaction during dyadic play, watching video feedback, or role-playing. One parent, for example, can keep a “still face” while the other tries to make contact, to enhance the empathy and understanding for the feeling of the child when the parent is emotionally unavailable. During a video feedback or real life interaction, another parent can “speak for the child.” Differentiating early attachment experiences, cultural influences, and the impact of trauma-induced influences on parental expectations, representations facilitate alternations in parental practices that interfere with positive parent–child relationships. Moreover, positive, and critical feedback from other parents is powerful and easier to accept.

### Regulation of emotions

Parents’ capacity to tolerate and regulate their own emotions, enhances the containment, and regulation of the emotional experiences of their children (63). Posttraumatic stress responses, limit the reflexive capacities of a traumatized parent. Hyperarousal, anger and irritability can lead to parental hostility or, in contrast, hypo-arousal and numbing of emotions can increase hostility and emotional unavailability, both interfering with parental sensitivity (59). Developing skills that enhance emotional regulation help parents to retain their optimal arousal. When parents learn how to recognize triggers and gain control over intense emotions that can be frightening for children and lead to neglect and hostility, they are also learning the process of bolstering secure attachment. Thus, even if the trauma treatment is not possible at the moment or might take a long time, the parents can still learn to regulate their emotions better, become emotionally more available, and avoid moments of harsh parenting. By strengthening their coping with intense emotions, parents can gradually become less preoccupied with their own problems and instead prioritize the children’s needs. In family group meetings, parents can exercise new behavior in a group in various settings and learn skills to meet the needs of their children, comfort, and set limits calmly when needed. Group members may be asked to critically support attempts of new behavior.

### Empowerment: Strengthening resilience

Resilience can be forged even when problems exist (45). Building up resilience is an ongoing process in MFT. The idea that families are helping each other while the therapist is in the background, encourages traumatized parents to feel less helpless and powerless, encourages them to activate their own resources and to overcome social isolation. Positive segments of video feedback, mutual support, and feedback, discovering and building on competencies are used to strengthen the self-esteem of parents and generate hope that despite internal and external problems they can become good-enough parents for their children. The ability to support others, to give the benefit of your experience to others, is rewarding. This may be significant to people who have lived with numerous difficulties for a long time. Chronic and multiple adversities undermine the feeling of efficacy and hope. Being successful in helping others may contribute to the start of feeling more resilient and powerful.

Playful activities with children, short moments of pleasure and joy, become signs for parents that change is possible and are helpful for building hope. Supporting parents to share coping strategies to deal with stress, discover and exchange their individual, family, and cultural resources strengthens parental resilience, and, in turn, helps parents to stimulate the adaptive potentials of the children.

### Families supporting each other

The multiple family setting with four to six families, creates holding, a secure and supportive base for isolated families. Families support each other and the therapist’s role is to facilitate and maximize interactions and create a context for change. Families supporting each other is one of the most fruitful ways of facilitating family adaptation and change in the face of adversity (64). Having four to six families with several members in a session implies the presence of extensive experience. Other group members are crucial both for support and feedback. When sensitive issues such as parenting are concerned, feedback of other parents prevents severe feelings of shame and failure, common in many traumatized parents. This can be fostered by working in subgroups, e.g., with individuals, their partners, or couples.

### Specific target groups: A multicultural setting

Working with traumatized families and their children from different political, religious, and cultural backgrounds, means to deal with multiple markers of diversity. This applies to all target groups, such as police officers, veterans, and refugees. Cultural, gender, political and religious influences can be intertwined with post-traumatic limitations of parenting in a variety of ways. Culture has been described as the prime context for determining associations between activity, such as parent–child interaction and meaning, influencing parental cognitions and practices (65). Even though virtually all aspects of parenting children are informed by culture, the underlying pattern of sensitivity as a healthy component to a parent–child interaction that is beneficial to the child is universal (66, 67). Therefore, specific parenting behaviors may vary across cultures, but the patterning of sensitive behavior remains the same. Parenting interventions focused on enhancing sensitivity fit the beliefs of mothers of young children in different cultural groups (68). Cultural adaptations of the program include sensitivity to the degree and influence of specific cultural family risk and protective factors, level and influence of acculturation, and migration history (69).

The influence of traumatic experiences and symptoms on parenting, or use of coping strategies, can be very different, depending on familial and cultural context. What seems logical though unhelpful to one family can be perceived from another perspective by others. Multifamily group therapy, with families arriving from different parts of the world such as West Africa, Caucasus, Balkan, or the Middle East, opens possibilities for an intercultural exchange of helpful strategies and generates many options to discuss—what their strengths are as a family, what gives them hope, and how they help each other. With MFT groups, both commonalities and differences or contrasts matter. Similarities between members help to feel acknowledged and increase a sense of safety and trust. Contrasts, moreover, are needed for the exchange of different opinions, thoughts, and ideas. Differences based on cultural background, living circumstances, age, or gender, help to challenge fixed ideas, prejudice, and assumptions. A curiosity for other members of the group is crucial for cohesion to occur. This parallels the attention and sensitivity parents need to have for their children (59).

## Conclusion

Recent research findings suggest multiple pathways of intergenerational processes that influence the quality of parent–child relationships and the development of children. Reduced sensitivity, responsivity, and increased hostility associated with posttraumatic symptoms of refugee parents are risk factors for secure attachment. Pre- and post-natal PTSD of mothers can cause alternations of the system that regulates stress, through neuro-endocrinal and genetical mechanisms, leading to higher vulnerability to post-traumatic stress disorder by children. Identified key issues in relational patterns such as mentalization, attachment, the cycle of violence, and physiological transmission are important in guiding interventions. The impact of parental trauma on children can be seen as a part of a dynamic systemic process of family adaptation including positive aspects of intergenerational dimensions of parental trauma that foster resilience. The risk and protective potentials of intergenerational influence of parental trauma by refugees need to be considered within the broader community and socio-cultural context in pre- and post-migration period.

The treatment of traumatized families aims to reduce PTSD and related parental limitations, enhance sensitive parenting, and secure attachment. Mentalization, emotional regulation, and empowerment are used as the main pathways to reduce trauma-related intergenerational effects on parenting, parent–child relationships, and child development. The treatment according to a group format (MFT) is multidimensional and focuses on the interplay between internal (biological, psychological) and external (familiar, cultural, social, political) processes that hinder or help the development of children and family. MFT creates context for change through variations of flexible settings (intra-family, inter-family, parents–children, individual) integrating parent–child interventions with trauma treatment of parents. The dynamic interplay of commonalities and differences within a multicultural group setting opens possibilities to generate multiple perspectives on the effects of traumatic experiences and PTSD symptoms on parenting. In a safe, holding group, families from various cultural backgrounds support each other in the joint search for how to become “good enough” parents for their children despite past traumatic experiences and ongoing stress.

## Author contributions

TM, EE, IH, and JB contributed to developing and sharing ideas for the manuscript, were very familiar with the work and treatment, and wrote segments of the manuscript. TM integrated all pieces. All authors contributed to the article and approved the submitted version.
